# Mitogen activated protein kinase kinase kinase 3 (MAP3K3/MEKK3) overexpression is an early event in esophageal tumorigenesis and is a predictor of poor disease prognosis

**DOI:** 10.1186/1471-2407-14-2

**Published:** 2014-01-02

**Authors:** Raghibul Hasan, Rinu Sharma, Anoop Saraya, Tushar K Chattopadhyay, Siddartha DattaGupta, Paul G Walfish, Shyam S Chauhan, Ranju Ralhan

**Affiliations:** 1 Department of Biochemistry, All India Institute of Medical Sciences, Ansari Nagar, New Delhi 110029, India; 2 School of Biotechnology, Guru Gobind Singh Indraprastha University, Kashmere Gate, Delhi 110403, India; 3 Department of Gastroenterology, All India Institute of Medical Sciences, Ansari Nagar, New Delhi 110029, India; 4 Department of Gastrointestinal Surgery, All India Institute of Medical Sciences, Ansari Nagar, New Delhi 110029, India; 5 Department of Pathology, All India Institute of Medical Sciences, Ansari Nagar, New Delhi 110029, India; 6 Department of Medicine, Endocrine Division, Mount Sinai Hospital and University of Toronto, Toronto, ON M5G 1X5, Canada; 7 Alex and Simona Shnaider Research Laboratory in Molecular Oncology, Department of Pathology & Laboratory Medicine, Mount Sinai Hospital, Toronto, ON M5G 1X5, Canada; 8 Department of Pathology and Laboratory Medicine, Mount Sinai Hospital, Toronto, ON M5G 1X5, Canada; 9 Joseph and Mildred Sonshine Family Centre for Head and Neck Diseases, Department of Otolaryngology – Head and Neck Surgery, Mount Sinai Hospital, Toronto, ON M5G 1X5, Canada; 10 Department of Otolaryngology – Head and Neck Surgery, University of Toronto, Toronto, ON M5G 2N2, Canada

**Keywords:** MEKK3, ESCC, Diagnosis, Dysplasia, Immunohistochemistry, Prognosis

## Abstract

**Background:**

Mitogen-activated protein kinase kinase kinase3 (MAP3K3/MEKK3) was identified to be differentially expressed in esophageal squamous cell carcinoma (ESCC) using cDNA microarrays by our laboratory. Here in we determined the clinical significance of MEKK3 in ESCC.

**Methods:**

Immunohistochemical analysis of MEKK3 expression was carried out in archived tissue sections from 93 ESCCs, 47 histologically normal and 61 dysplastic esophageal tissues and correlated with clinicopathological parameters and disease prognosis over up to 7.5 years for ESCC patients.

**Results:**

MEKK3 expression was significantly increased in esophageal dysplasia and ESCC in comparison with normal mucosa (p_trend_ < 0.001). Kaplan Meier survival analysis showed significantly reduced median disease free survival median DFS = 10 months in patients with MEKK3 positive ESCCs compared to patients with no immunopositivity (median DFS = 19 months, p = 0.04). ESCC patients with MEKK3 positive and lymph node positive tumors had median DFS = 9 months, as compared to median DFS = 21 months in patients who did not show the alterations (p = 0.01). In multivariate Cox regression analysis, combination of MEKK3 overexpression and node positivity [p = 0.015, hazard ratio (HR) = 2.082, 95% CI = 1.154 - 3.756] emerged as important predictor of reduced disease free survival and poor prognosticator for ESCC patients.

**Conclusions:**

Alterations in MEKK3 expression occur in early stages of development of ESCC and are sustained during disease progression; MEKK3 in combination with lymph node positivity has the potential to serve as adverse prognosticator in ESCC.

## Background

Esophageal cancer is among the ten most common cancers worldwide and the sixth most common cause of death from cancer [1,2]. The patients with this malignancy have extremely poor prognosis owing to insidious symptomatology, late clinical presentation and rapid progression [3]. Esophageal squamous cell carcinoma (ESCC) is the major histological subtype of esophageal cancer, being the second most common cancer among males and the fourth most common cancer among females in India [4]. Despite advances in multimodality therapy, due to late stage of diagnosis and poor efficacy of treatment, the average 5-year survival rate for ESCC patients is about 30% globally [5-7]. Development of better preventive and diagnostic approaches as well as more effective treatment modalities requires an in-depth understanding of molecular mechanisms implicated in the complex process of esophageal carcinogenesis. Despite considerable diagnostic and therapeutic advances in the management of ESCC in recent years there still remains an urgent need for identification of novel molecular markers to provide the clinician with useful information concerning patient prognosis and possible therapeutic options [8-16]. In search of molecular markers our laboratory analyzed global gene expression profiles of ESCCs, using commercially available 19.1 k cDNA microarrays. MEKK3 cDNA was one of the lead found to be overexpressed in ESCCs [17]. These findings were verified using real-time quantitative RT-PCR analysis that showed significant increase in expression of MEKK3 transcripts in dysplasia and ESCCs as compared to normal esophageal tissues [17].

The mitogen-activated protein kinases (MAPKs) are a family of serine/threonine kinases that play important regulatory roles in a wide variety of biological processes [18]. Numerous mitogen-activated protein 3 kinases (MAP3Ks) have been identified, including MEKK1, MEKK2, MEKK3, MEKK4, tumor progression locus 2, and transforming growth factor-B-activated kinase 1, that are activated by linear phosphorylation cascades. MAP3Ks are emerging as important regulators of nuclear factor kappa B (NF-κB). MEKK3 is also called MAP3K3, a kinase capable of activating both the ERK and the stress-activated protein kinase cascades. It is positioned upstream of SEK and MEK in the signalling pathways and directly phosphorylates these enzymes. Overexpression of MEKK3 has been reported to occur frequently in ovarian cancer [19] that leads to increased NF-κB activity and increased expression of cell survival factors which ultimately contributes to their resistance to apoptosis. In contrast, MEKK3 has been demonstrated to be required for endothelium function but is not essential for tumor growth and angiogenesis [20]. These reports clearly emphasize the need for in depth investigations of the clinical relevance of MEKK3 in human cancers. The aim of the present study was to examine the clinical significance of MEKK3 in ESCC and determine the correlation between MEKK3 expression and clinicopathological parameters of ESCC patients. Further, we aimed to assess the prognostic relevance of MEKK3 in ESCC patients.

## Methods

### Patients and clinicopathological data collection, tissue specimens

The Institutional Human Ethics Committee of the All India Institute of Medical Sciences (AIIMS), New Delhi, India, approved this study prior to its commencement. Tissue specimens were obtained by diagnostic or therapeutic procedures from patients with clinically defined esophageal dysplasia (n = 61) attending the Outpatient Clinic of the Departments of Surgical Disciplines and Gastrointestinal Surgery, AIIMS. Tissue specimens were also collected from 93 ESCC patients undergoing curative cancer surgery during the period 2005–2010, after obtaining the patients’ written consent. Wherever possible, non-malignant tissues were taken, each from a site distant from the surgically resected ESCC. Non-malignant esophageal tissues were also collected from the patients attending the Endoscopy clinic in the Outpatient Department of Gastroenterology, after obtaining the patients’ written consent. Taken together, these 47 non-malignant esophageal tissues with histological evidence of normal epithelia constituted the normal group. After excision, tissues were immediately snap-frozen in liquid nitrogen and stored at −80°C in the Research Tissue Bank till further use; one part of the tissue was collected in 10% formalin and embedded in paraffin for histopathological and immunohistochemical analyses. Histologically confirmed esophageal normal epithelia, dysplasia, and ESCC as revealed by hematoxylin and eosin (H&E) staining were used for immunohistochemistry [21,22]. Patient demographic, clinical, and pathological data were recorded in a pre-designed Performa as described previously to establish a clinical database [21,22]. The information documented included clinical TNM staging (tumor, node, and metastasis based on the Union International Center le Cancer TNM classification of malignant tumors 2002), site of the lesion, histopathological differentiation, age and gender. All the ESCC tissues analyzed in this study had more than 80% tumor cells in H&E sections.

### Follow-up study

Eighty two of 93 ESCC patients who underwent treatment from 2005–2010 could be investigated and evaluated in the esophageal cancer follow-up clinic at regular time intervals, while 11 patients did not report in the follow up clinic. Survival status of the ESCC patients was verified and updated from the records of the Tumor Registry, Department of Gastrointestinal Surgery, AIIMS, as of June 2013. ESCC patients were monitored for a maximum period of 7.5 years. Disease-free survival time is defined as the time from completion of primary treatment till the patient showed any clinical and radiological evidence of local or regional disease, or distant metastasis at the time of the last follow-up of patients monitored in this study. Thirty one patients who did not show recurrence were alive until the end of the follow-up period. Only disease-free survival (expressed as the number of months from the date of surgery to loco-regional relapse/death) was evaluated in the present study, as the number of deaths due to disease progression did not allow a reliable statistical analysis.

### Immunohistochemistry

Paraffin-embedded sections (5 μm) of human esophageal histological normal (n =47), dysplasia (n = 61) and ESCC (n = 93) were collected on gelatin-coated slides. In brief, the sections were deparaffinized in xylene, hydrated in gradient alcohol, and pre-treated in a microwave oven for 10 min at 800 W and 5 min at 480 W in Citrate buffer (0.01 M, pH = 6.0) for antigen retrieval. The sections were incubated with hydrogen peroxide (3% v/v) in methanol for 30 min to quench the endogenous peroxidise activity, followed by blocking with 1% bovine serum albumin (BSA) to preclude non-specific binding. Thereafter, the slides were incubated with rabbit polyclonal anti-MEKK3 antibody (0.5 mg/ml, sc-28769, Santa Cruz Biotechnology, San Diego, CA) for 16 h at 4°C. The primary antibody was detected using the streptavidin-biotin complex with the Dako LSAB plus kit (Dako Cytomation, Glostrup, Denmark) and diaminobenzidine as the chromogen as described previously [23]. In the negative control tissue sections, the primary antibody was replaced by isotype specific non-immune mouse IgG. A section from breast cancer tissue was used as a positive control in each batch of immunohistochemistry.

### Evaluation of immunohistochemical staining

Each tissue section was evaluated for MEKK3 immunostaining using a semi-quantitative scoring system for both staining intensity and the percentage of positive epithelial cells [22]. For MEKK3 protein expression, sections were scored as positive if epithelial cells showed immunopositivity in the nucleus/cytoplasm when observed independently by three of us (MRH, RS, SDG), who were blinded to the clinical outcome (the slides were coded and the scorers did not have prior knowledge of the local tumor burden, lymphonodular spread, and grading of the tissue samples). The tissue sections were scored based on the% of immunostained cells as: ≤10% = 0; 11–30% = 1; 31–50% = 2; 51–70% = 3 and > 70% = 4. Sections were also scored semi-quantitatively on the basis of staining intensity as negative = 0; mild = 1; moderate = 2; intense =3. Finally, a total score was obtained by adding the scores of percentage positivity and intensity. The scoring by the three observers was discrepant in about 5% cases and a consensus on the final result was reached by re-evaluation of these slides and discussion. Based on sensitivity and specificity values for MEKK3, a total score cut-off value of 3 was defined as MEKK3 immunopositivity.

### Statistical analyses

The immunohistochemical data were subjected to statistical analyses using the SPSS 13.0 software (Chicago, IL). Sensitivity and specificity were calculated and quantified using receiver operating characteristic (ROC) analyses. The positive predictive value (PPV) describes the proportion of the correctly classified cases. A total score cut-off value of 3 was defined as MEKK3 immunopositivity for statistical analyses. The relationships between MEKK3 protein expression and clinicopathological parameters were tested using Chi-Square and Fischer’s exact test. Two-sided p values were calculated and p < 0.05 was considered to be significant. Similarly, PPV was calculated for esophageal dysplasia and ESCC with respect to normal tissues. The correlation of MEKK3 staining with patient survival was evaluated using life tables constructed from survival data with Kaplan-Meier plots. Multivariate analysis was carried out using Cox regression model [24].

## Results

### Immunohistochemical analysis of MEKK3 expression in esophageal normal, dysplasia and cancer

**Figure 1:**
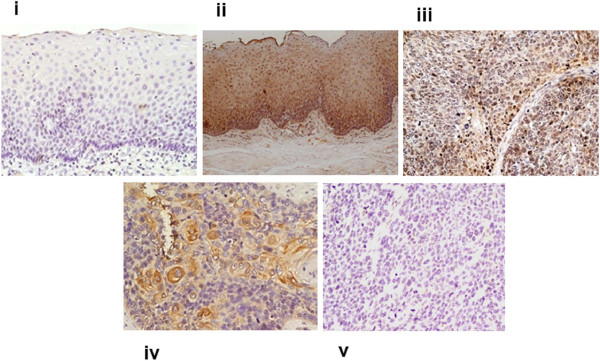
**Immunohistochemical analysis of MEKK3 in esophageal tissues.** Paraffin-embedded sections of histologically normal mucosa, dysplasia, and ESCC were stained using anti-MEKK3 polyclonal antibody as described in the Methods section. **(i)** Normal esophageal mucosa showing no MEKK3 immunostaining; **(ii)** dysplasia depicting nuclear and cytoplasmic MEKK3 immunostaining in epithelial cells; **(iii)** ESCC illustrating both intense cytoplasmic and nuclear staining in tumor cells; **(iv)** ESCC section showing cytoplasmic MEKK3 immunostaining; **(v)** ESCC used as a negative control incubated with isotype specific IgG replacing the primary antibody showing no MEKK3 immunostaining in tumor cells (**(i-v)** original magnification x 200).

**Figure 2:**
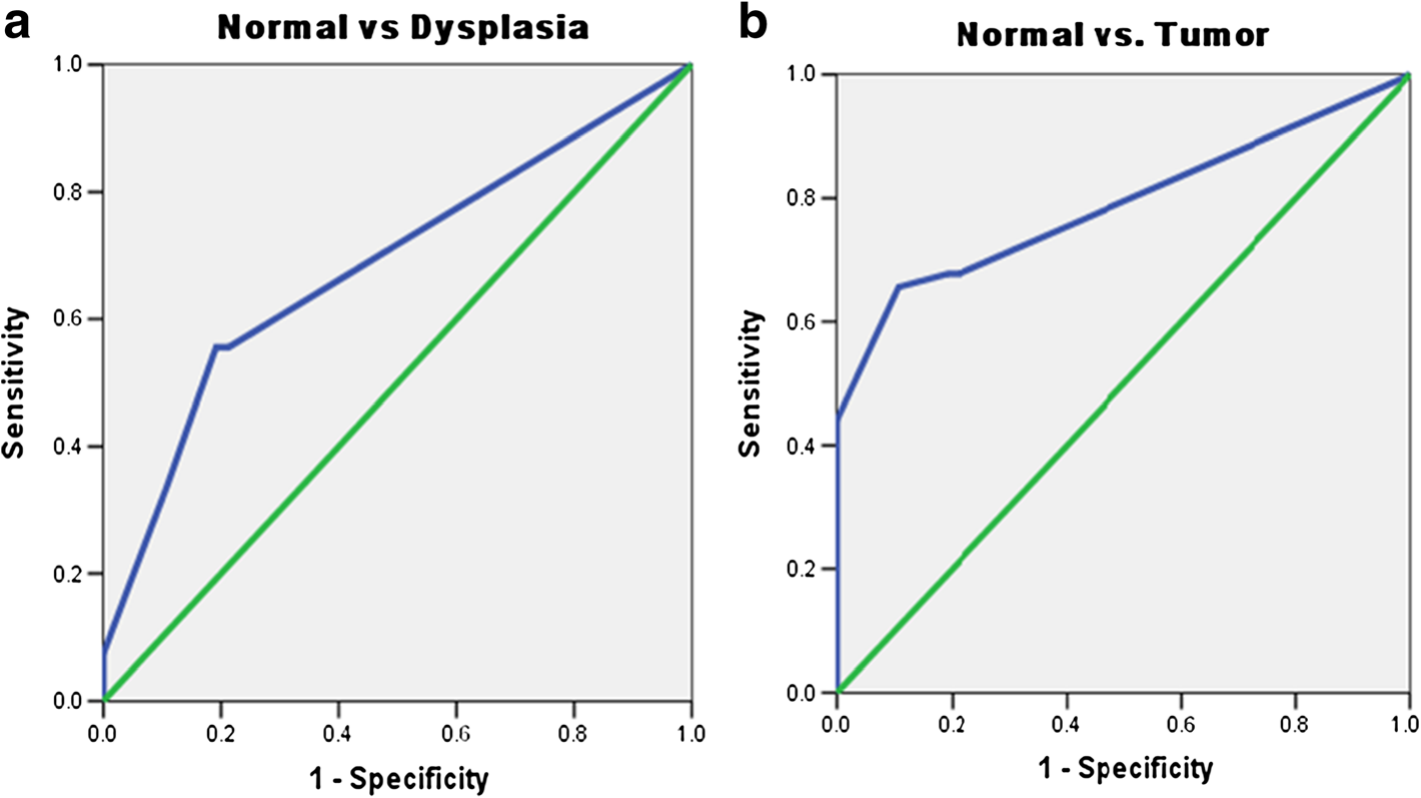
**Receiver operating curve (ROC) analysis for MEKK3 expression in esophageal tissues. (a)** Normal versus Dysplasia; **(b)** Normal versus Cancer.

To determine the clinical significance of MEKK3 protein in ESCC, its expression was analyzed in clinical specimens from, histologically normal esophageal tissues, dysplasia, and ESCC using a specific anti-MEKK3 antibody by immunohistochemistry. Of the 47 normal tissues analyzed, 37 (79%) cases did not show detectable MEKK3 immunostaining in nucleus/cytoplasm of epithelial cells [Figure 1 (i)]; moderate staining was observed in differentiated epithelial cells in 10/47 (21%) normal tissues. Chi square trend analysis showed significant increase in MEKK3 expression (cytoplasmic/nuclear) in tissues obtained from different stages of esophageal tumorigenesis (normal, dysplasia and ESCC; Table 1, p_trend_ < 0.001). Notably, significant increase in cytoplasmic/nuclear localization of MEKK3 was observed in 34 of 61 (55.7%) dysplasia cases (p < 0.001, odd’s ratio (OR) = 4.6, 95% CI = 1.9-11.0) compared to normal esophageal tissues [Table 1 and Figure 1 (ii)]. Similar localization pattern of MEKK3 immunostaining was observed in ESCC as well [Figure 1 (iii, iv)]. Sixty three of 93 (67.7%) ESCCs showed cytoplasmic/nuclear localization of MEKK3 in tumor cells as compared to the normal tissues (p < 0.001, OR = 7.77, 95%, CI = 3.41-17.7). However, no significant correlations were observed between clinicopathological parameters of ESCC and MEKK3 expression (Table 1). No immunostaining was observed in ESCC tissue sections used as negative controls where the primary antibody was replaced by isotype specific IgG (Figure 1v). Receiver Operating Characteristic (ROC) analysis was used to determine the area-under-the-curve (AUC) - 0.68 and 0.80, with sensitivity of 55.56% and 52.46% for dysplasia and ESCC respectively, and specificity of 78.72% for both (Figure 2A and B; Table 2).

**Table 1:** Immunohistochemical analysis of MEKK3 protein in esophageal tissues and relationship with clinicopathological parameters

**Clinicopathological features**	**Total cases (N)**	**Cytoplasmic/nuclear positivity n (%)**	**P-value**	**OR (95% CI)**
Normal	47	10 (21)		
Dysplasia	61	34 (55.7)	**<0.001**	**4.6 (1.9-11.0)**
ESCC	93	63 (67.7)	**0.001**	**7.77 (3.41-17.7)**
Age (years)				
<54	43	30 (69.8)	0.825	0.84 (0.35-2.01)
> = 54	50	33 (66)		
Gender				
Male	62	40 (64.5)	0.481	1.58 (0.60-4.12)
Female	31	23 (74.2)		
Tumor stage				
(T_1_ + T_2_)	10	6 (60.0)	0.722	1.46 (0.38-5.62)
(T_3_ + T_4_)	83	57 (68.7)		
Nodal status				
N_0_	27	19 (70.4)	0.81	0.84 (0.31-2.22)
N_1_	66	44 (66.7)		
Histopathology				
WDSCC	30	20 (66.7)	0.61	0.6 (0.20-1.87)
MDSCC	48	33 (68.7)		
PDSCC	15	10 (66.7)		

**Table 2:** Biomarker analysis of MEKK3 expression in esophageal tissues

**MEKK3**	**Sensitivity**	**Specificity**	**PPV**	**AUC**
Normal vs. dysplasia	55.56	78.72	60.0	0.68
Normal vs. ESCC	52.46	78.72	86.3	0.80

### MEKK3 overexpression as a prognostic marker for ESCC

**Figure 3:**
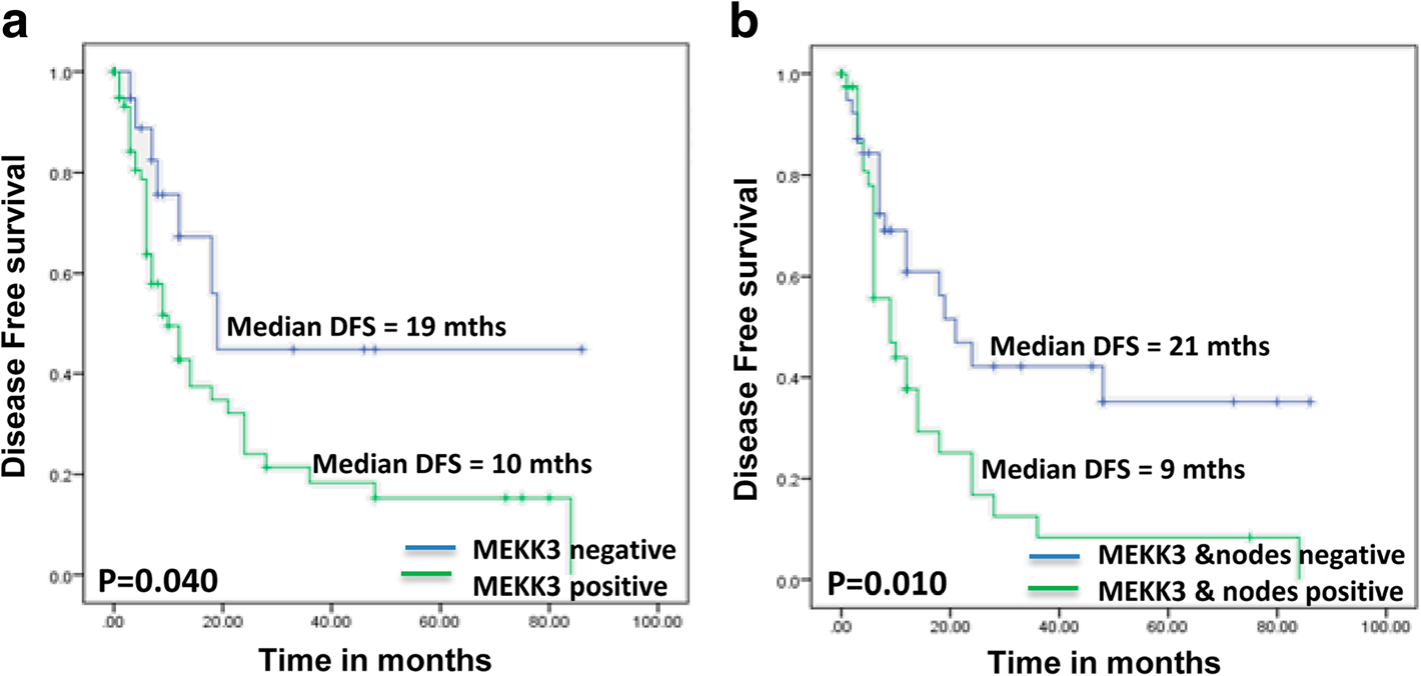
**Evaluation of MEKK3 overexpression as a prognostic marker in ESCC.** Kaplan–Meier estimation of cumulative proportion of disease-free survival: **(a)** Median time for disease-free survival (DFS; no recurrence/metastasis) in patients showing increased MEKK3 expression (shown with solid line) DFS was 10 months and was significantly reduced as compared to patients with ESCC that showed no or mild immunostaining (p = 0.005; median DFS = 19 months, shown with dotted line); **(b)** Median time for disease-free survival (DFS; no recurrence/metastasis) in patients showing increased MEKK3 expression and lymph node positivity (shown with solid line) DFS was 9 months and was significantly reduced as compared to patients with ESCC that showed no or mild immunostaining (median DFS = 21 months; range 1–90 months p = 0.01; shown with dotted line).

Kaplan–Meier survival analysis showed significantly reduced disease-free survival (median disease free survival = 10 months) in ESCC patients harbouring increased MEKK3 expression compared with the patients showing no nuclear/cytoplasmic MEKK3 immunostaining (p = 0.04, median DFS = 19 months), (Figure 3a). Notably, ESCC patients showing nodal positivity and MEKK3 overexpression had significantly reduced median DFS = 9 months in comparison node negative patients with low MEKK3 expression (median DFS = 21 months; range 1–90 months; p = 0.01) (Figure 3b).

Cox regression analysis was carried out to determine the prognostic potential of MEKK3 overexpression for ESCC in comparison with the other clinical parameters - nodal status (Table 3). MEKK3 overexpression in combination with nodal metastasis emerged as the most significant prognostic marker for ESCC (p = 0.015 HR = 2.082, 95% CI = 1.154- 3.756) (Table 3).

**Table 3:** Correlation of overall survival with clinicopathological parameters and MEKK3 expression: multivariate analysis

**Clinico- pathological parameter**	**Kaplan Meier Survival analysis**	**Multivariate Cox regression analysis**	**Hazard ratio (95% CI)**
	**Un-adjusted p-value**	**Adjusted p-value**	
MEKK3 and lymph node positivity	**0.010**	**0.015**	2.082 (1.154- 3.756)
MEKK3 Staining	**0.040**	0.379	

## Discussion and conclusions

Our study demonstrated significant increase in MEKK3 expression in esophageal dysplasia and ESCC in comparison with normal esophageal tissues. Further, correlation of MEKK3 expression with clinical outcome showed that overexpression of this protein is associated with shorter disease free survival and thus poor prognosis of ESCC patients. Our findings are important in view of the fact that studies on molecular analysis of esophageal dysplasia are very limited, often because these patients do not seek medical attention due to small size of the lesions that do not pose any serious clinical problems, or patients avoid endoscopic examination. Therefore, there are no established biomarkers that can be used in clinics routinely in early stages of the disease. The histological evidence of dysplasia is insufficient to identify lesions that are at high risk of cancer development. Hence, overexpression of MEKK3 observed in dysplastic lesions is an important finding of our study that underscores its potential as an early marker. Notably, MEKK3 protein was detected in the distant dysplastic esophageal tissues in patients with ESCC, while no detectable expression was observed in the matched histologically normal esophageal epithelia distant from the tumors. The presence of MEKK3 in early preneoplastic lesions, and localized expression of MEKK3 in areas of high proliferative activity support the hypothesis that alteration in MEKK3 expression is an early event in esophageal tumorigenesis.

The hallmark of the study was the detection of MEKK3 protein in endoscopic biopsies of esophageal epithelial dysplasia, suggesting its potential for development as an early biomarker. We are cognizant of the fact that limitations of our study are the small size of dysplasia cases investigated and lack of follow-up data of patients with dysplasia. Nevertheless, to our knowledge this is the first study demonstrating overexpression of MEKK3 in early stage prior to development of frank malignancy, as well as in ESCC, that offers an opportunity for early detection and intervention for effective management of this disease, which otherwise has poor prognosis (overall 5-year survival ranges from 15-25%) particularly when detected in late stages [25]. Our findings warrant long term longitudinal follow-up studies of patients with esophageal dysplasia that go on to develop ESCC, to establish a possible link between MEKK3 overexpression and risk of cancer development. Nevertheless, in view of the lack of availability of molecular markers for early diagnosis of ESCC having insidious symptomatology, our findings are of potential clinical relevance. The molecular basis of MEKK3 overexpression in esophageal dysplasia is supported by the recent review on the role of cancer–related inflammation in ESCC [26].

The significant increase in MEKK3 expression observed in ESCC (67.7% cases) as compared to normal esophageal tissues is another important finding of our study suggesting that accumulation of MEKK3 may be linked to increased risk of malignant transformation and might serve as a marker to identify the high-risk lesions. Notably, our study suggested the clinical significance of MEKK3 overexpression as a predictor of poor prognosis of ESCC. To our knowledge this the first report of MEKK3 as a biomarker of prognostic relevance in ESCC. The functional significance of MEKK3 protein in esophageal tumorigenesis remains to be determined. We speculate that with a growing understanding of the role of MEKK3 in cell migration, invasion and proliferation pathways, the potential of MEKK3 as a therapeutic target for the treatment of cancer should be the subject of future studies. In support of our findings MEKK3 overexpression has been reported in ovarian cancer as compared to normal ovarian epithelial cells [19].

In conclusion, this study provides evidence of MEKK3 overexpression in dysplastic esophageal epithelium as well as in ESCC, suggesting that MEKK3 expression is altered in early stages and sustained in esophageal tumorigenesis. These findings are of immense clinical relevance in view of the fact that early detection of ESCC is severely hampered by the paucity of molecular markers for diagnosis of this aggressive malignancy in initial stages. MEKK3 overexpression is a predictor of poor prognosis of ESCC. Furthermore, increased accumulation of MEKK3 in ESCC as compared to dysplastic lesions warrants a large-scale longitudinal study of patients with dysplasia to evaluate its potential as a determinant of increased risk of progression to cancer and as a marker for recurrence of ESCC.

## Abbreviations

CI: Confidence interval; DFS: Disease free survival; ESCC: Esophageal squamous cell carcinoma; FFPE: Formalin fixed and paraffin embedded; HR: Hazard ratio; H&E: Hematoxylin and eosin; IHC: Immunohistochemical analysis; MEKK3: Mitogen-activated protein kinase kinase kinase3.

## Competing interests

The authors declare that they have no competing interests.

## Authors’ contributions

MRH carried out the experimental work, data analysis and drafted the manuscript. MRH, RS and SDG evaluated the H&E stained and immunostained slides. AS and TKC provided clinical specimens for this study, clinical perspective and follow-up data. RR, SSC, RS and PGW conceived the study, participated in its design and coordination, provided infrastructural and financial support and edited the manuscript. All authors read and approved the final manuscript. RR and SSC provided infrastructural and financial support for this study.

## Pre-publication history

The pre-publication history for this paper can be accessed here:

http://www.biomedcentral.com/1471-2407/14/2/prepub
